# Topical Application of TGF-β-Activating Peptide, KRFK, Prevents Inflammatory Manifestations in the TSP-1-Deficient Mouse Model of Chronic Ocular Inflammation

**DOI:** 10.3390/ijms20010009

**Published:** 2018-12-20

**Authors:** Laura Soriano-Romaní, Laura Contreras-Ruiz, Antonio López-García, Yolanda Diebold, Sharmila Masli

**Affiliations:** 1 Ocular Surface Group, IOBA—University of Valladolid, 47011 Valladolid, Spain; lsoriano@ioba.med.uva.es or laurasorianoromani@gmail.com (L.S.-R.); antonio.lopez@ioba.med.uva.es (A.L.-G.); 2 Department of Ophthalmology, Boston University School of Medicine, Boston, MA 02118, USA; lcontrerasruiz15@gmail.com; 3 Biomedical Research Networking Center on Bioengineering, Biomaterials and Nanomedicine (CIBER-BBN), 28029 Madrid, Spain

**Keywords:** Inflammation, KRFK peptide, ocular surface, thrombospondin-1, transforming growth factor-β

## Abstract

Chronic inflammation of the ocular surface poses a risk of vision impairment. The understanding of the molecular mechanisms that are involved in the inflammatory response is critical to identify novel molecular targets. Recently, thrombospondin-1 (TSP-1) has emerged as a key player in ocular surface homeostasis that efficiently activates the TGF-β2 isoform that is predominantly expressed in the ocular mucosa. Here, the potential of the peptide derived from TSP-1 (KRFK), that can activate TGF-β, is proposed as a potentially applicable therapeutic for chronic ocular surface inflammatory disorders. Our in vitro results confirm that the chosen peptide activates TGF-β, reducing the expression of co-stimulatory molecules on dendritic cells, driving them towards a tolerogenic phenotype. For the in vivo studies, the TSP-1^−/−^ mouse is used as a pre-clinical model of chronic ocular inflammation. We observe that the topical application of KRFK alters the peripheral balance of effectors by reducing the proportion of pathogenic Th1 and Th17 cells while increasing Treg cell proportion in cervical lymph nodes. In line with these findings, the development of chronic ocular surface inflammation is significantly prevented in KRFK-treated TSP-1^−/−^ mice, as assessed by clinical parameters and inflammatory cytokine expression in conjunctival and lacrimal gland tissues. Together, our results identify the KRFK peptide as a novel therapeutic option to prevent the development of chronic inflammatory manifestations of the ocular surface.

## 1. Introduction

Chronic ocular surface inflammatory diseases can cause the loss of visual acuity, quality of life, and may be accompanied by pain. At present, treatments for chronic ocular inflammatory diseases predominantly include long-term use of steroids or immunosuppressive drugs, both being known to cause undesirable side effects. There is a pressing need to develop improved and effective therapeutic approaches that target underlying mechanisms.

The glycoprotein thrombospondin (TSP)-1 is a key player in mucosal immune homeostasis at the ocular surface due to its ability to modulate local antigen-presenting cell (APC) phenotype towards an immature state. It is known that TSP-1 signaling through CD47 receptor induces a tolerogenic phenotype of dendritic cells [1,2]. It is also known to modulate ocular APC phenotype through activation of the predominant isoform of transforming growth factor β (TGF-β) in the ocular surface: the TGF-β2 [3,4,5].

The absence of such APC regulation in TSP-1-deficient mice explains the spontaneous development of chronic inflammatory ocular disease that is also accompanied with a peripheral imbalance between inflammatory and regulatory effector T cells [6]. Overall, these data support the relevance of TSP-1 in maintaining immune homeostasis at the ocular mucosa.

In subjects with chronic inflammatory ocular surface, disease reduced expression of TSP-1 is noted in conjunctival epithelial cells [7], while the increased expression of TGF-β2 is detected both in human subjects with ocular inflammation and the mouse model with induced inflammation [8,9]. Upfront, these results may appear paradoxical while considering the anti-inflammatory role of TGF-β. However, in mice with ocular surface inflammation, increased TGF-β2 was detected along with a reduced detection of TSP-1 receptor (CD36). Therefore, it is highly likely that the detected TGF-β2 is in its latent form and the absence of its biological activity corresponds with the inflammation.

While regulation of immune homeostasis by TGF-β isoforms, TGF-β1 and 3, depends on their activation by integrins that bind to Arg-Gly-Asp (RGD) sequences that are located in their latency associated peptide (LAP), TGF-β2 LAP does not contain the RGD sequence and it cannot be activated by integrins [10]. Therefore, TSP-1-mediated activation is likely to be critical in tissues like ocular mucosa without predominant TGF-β2 expression. A specific amino acid sequence, Arg-Phe-Lys (RFK), located between the first and the second type 1 TSR repeats of TSP-1 molecule, is identified as the minimal sequence that activates all isoforms of latent TGF-β [11].

In the ocular mucosa, TSP-1 regulates the local APC phenotype, which is crucial for maintaining the peripheral immune balance, and subsequently ocular surface health. Therefore, the use of peptides that are derived from TSP-1 may represent a potential therapeutic approach to treat ocular surface inflammatory diseases. Recently, a TSP-1-derived peptide that binds CD47 proved to reverse ocular surface inflammatory signs in TSP-1-deficient mice by promoting regulatory T cell (Treg) induction and inhibiting Th17 lymphocyte development [12]. Another TSP-1-derived peptide, Lys-Arg-Phe-Lys (KRFK), is known to bind Leu-Ser-Lys-Leu (LSKL) sequence within the LAP of latent TGF-β [13]. The interaction between LSKL and Arg-Lys-Pro-Lys (RKPK) sequence within the mature TGF-β is necessary to maintain TGF-β in its inactive/latent form. By competing for the LSKL binding, KRFK is known to activate latent TGF-β releasing its mature/active form that binds TGF-β receptors on target cells. Thus, the KRFK peptide is expected to facilitate TGF-β-mediated signaling and its downstream effects independently of TSP receptors, like CD47 and CD36. Application of the KRFK peptide has been reported to successfully activate latent TGF-β in mice [14]. However, the effect of restoring TSP-1-mediated activation of TGF-β on chronic ocular inflammation has not been tested yet and also achieving such activation via instillation in the form of eye drops has not been reported. In this study, we address this possibility and examine its effect on the development of chronic ocular inflammation given that eye drops represent the most common and preferred non-invasive method for drug administration for ocular surface disorders.

We test the hypothesis that if topically applied KRFK peptide overcomes ocular surface pharmacological barriers, activates TGF-β2 in the ocular mucosal tissue and facilitates the modulation of local dendritic cell (DC) phenotype, it will restore peripheral immune regulation and prevent ocular surface inflammation. Therefore, we determine whether topically administered TSP-1-derived peptide, KRFK, can prevent inflammatory signs in cornea, conjunctiva, and lacrimal glands of TSP-1-deficient mice.

## 2. Results

### 2.1. The KRFK Peptide Activates Secreted TGF-β and Reduces the Expression of DC Maturation Markers in TSP-1-Deficient Bone Marrow-Derived Dendritic cells (BMDCs) In Vitro

**Figure 1:**
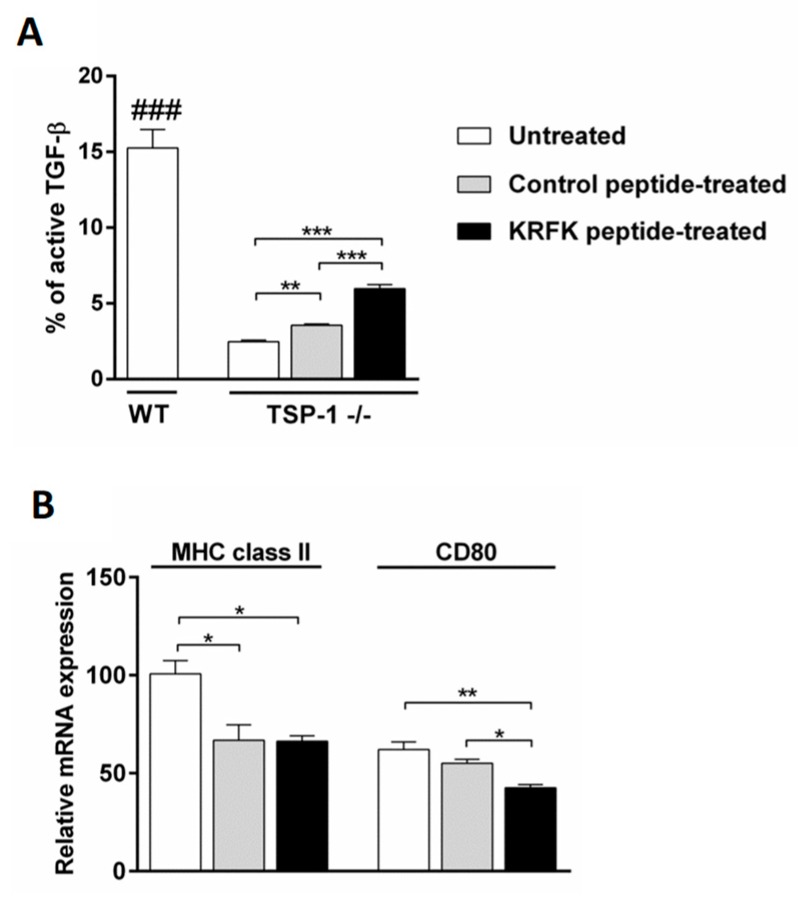
The KRFK peptide activates latent transforming growth factor β (TGF-β) produced by thrombospondin-1 (TSP-1)-deficient dendritic cells, modulating its phenotype in vitro. Wild type (WT) and TSP-1-deficient bone marrow dendritic cells (BMDCs) were cultured and treated with KQFK (control) or peptide derived from TSP-1 (KRFK). (**A**) The percentage of active TGF-β in the supernatants was determined. (**B**) Relative expression of MHC class II and co-stimulatory molecule CD80 was assessed in untreated and peptide-treated TSP-1-deficient BMDCs. Statistically significant differences between samples are indicated with asterisks (* *p* ≤ 0.05, ** *p* ≤ 0.01, *** *p* ≤ 0.001). WT values are significantly different compared to all other samples, as indicated by hashes (### *p* ≤ 0.001).

The amino acid sequence RFK present in the TSP-1 molecule can activate latent TGF-β [15]. Here we test if KRFK peptide can facilitate the activation of the endogenous latent TGF-β derived from TSP-1-deficient BMDCs that is otherwise not activated [4]. We used untreated wild type Bone Marrow-Derived Dendritic cells (WT BMDCs) as control. Culture supernatants from TSP-1-deficient BMDCs cultured in the presence of KRFK or inactive control (KQFK) peptides were analyzed for the content of total and inactive TGF-β levels. Percentage of active TGF-β as compared to total TGF-β is shown in Figure 1A. As expected, culture supernatants that were collected from WT BMDCs contained significantly higher proportion of active TGF-β as compared to that detected in culture supernatants of TSP-1-deficient BMDCs. However, in the presence of the KRFK peptide, this proportion of active TGF-β was significantly increased in supernatants of TSP-1-deficient BMDCs as compared to control cultures that were treated with inactive peptide (KQFK). These results support the ability of the KRFK peptide to activate endogenous latent TGF-β produced by BMDCs.

Among the immunoregulatory activities of TGF-β, is its ability to maintain DCs in an immature and tolerogenic state that is characterized by the low expression of MHC class II and co-stimulatory molecules. When considering the ability of the KRFK peptide to activate endogenous latent TGF-β produced by BMDCs, we evaluated whether this leads to modulation of BMDC phenotype. We compared the expression of DC maturation markers, MHC class II and CD80, by real-time PCR between untreated, KRFK, and control peptide treated TSP-1-deficient BMDCs. As shown in Figure 1B, the expression of both MHC class II and CD80 was down regulated in KRKF-treated BMDCs as compared to untreated controls. While the expression of MHC class II in control peptide-treated BMDCs was also down-regulated, their CD80 expression remained unaltered. Together, these results suggest that endogenous latent TGF-β from DCs activated by KRFK can indeed prevent their maturation.

### 2.2. Topically Administered KRFK Peptide is Retained in Ocular Surface Tissues

**Figure 2:**
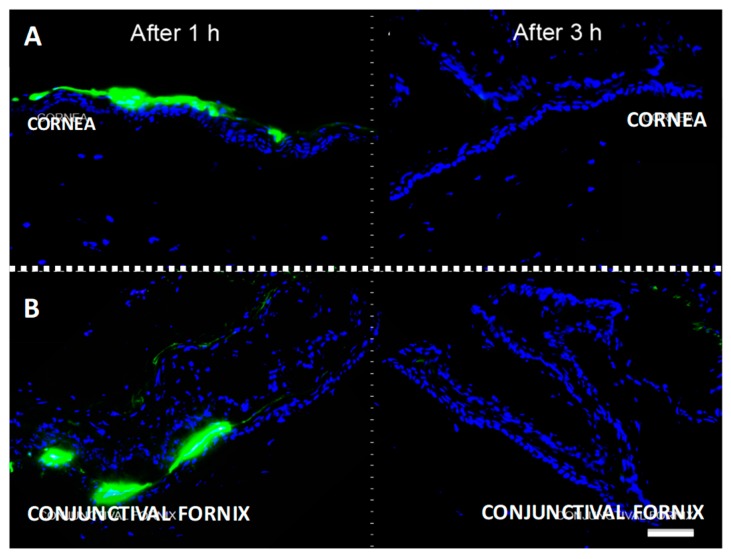
Topically administered fluorescein isothiocyanate (FITC)-KRFK peptide is retained at the ocular surface up to one hour. Ocular tissues were harvested after application of FITC-labeled KRFK peptide at 1 or 3 h intervals. Representative micrographs of the mouse cornea (**A**) and conjunctiva (**B**) are shown. FITC-KRFK (green) and DAPI-stained nuclei (blue). Scale bar: 50 μm.

We next address the in vivo effect of KRFK in mice. To achieve this, we first evaluated the potential dosing schedule of the KRFK in vivo. When considering the presence of DCs in the ocular surface tissues, cornea, and conjunctiva, we determined the retention time of topically administered KRFK at these sites. In these experiments, fluorescein isothiocyanate (FITC)-conjugated KRFK peptide was administered topically in mice and ocular surface tissues were harvested at 1 and 3 h intervals. Fresh frozen tissue sections were examined for the presence of FITC-conjugated KRFK. Results showed an intense FITC-fluorescence in areas of the cornea and the conjunctiva after 1 h of peptide application, demonstrating that the peptide was still within these ocular surface tissues (Figure 2). Particularly in the cornea, intense fluorescence was primarily in the outermost epithelial layer. However, in the conjunctiva, some peptide agglomeration was detected in the fornix area along with the presence of fluorescence signal in the stroma, providing evidence that topically applied KRFK is able to cross conjunctival the epithelial barrier. By three hours, no significant fluorescence was detectable in either of the ocular surface tissues. These results indicate the retention of topically applied KRFK peptide in the ocular surface tissues for at least 1 h, suggesting a possible modulation of local DC phenotype mirroring its in vitro effect.

### 2.3. Topically Administered KRFK Peptide to TSP-1-Deficient Mice Alters Peripheral Balance of CD4^+^ Inflammatory Effectors

**Figure 3:**
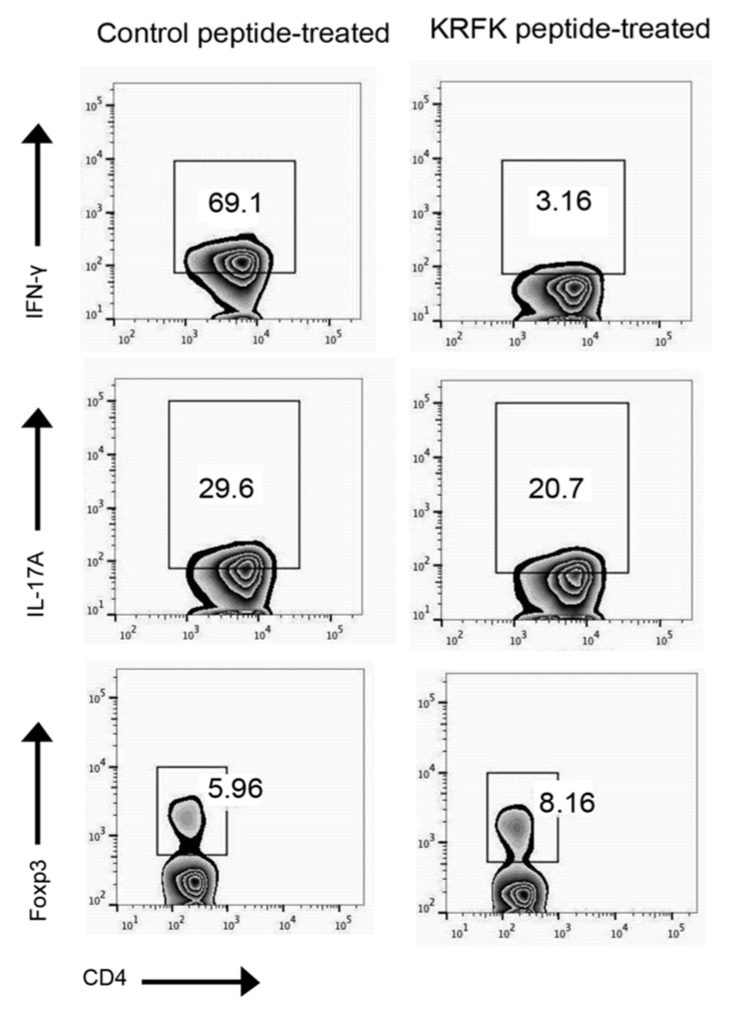
Topically administered KRFK peptide modulates effector phenotype in the draining lymph nodes of TSP-1-deficient mice. Flow cytometric analysis of phorbol 12-myristate 13-acetate (PMA)/ionomycin-stimulated lymph node cells harvested from peptide treated TSP-1 deficient mice were analyzed for intracellular IFN-γ, IL-17A, and Foxp3 staining in viable CD4^+^ cells. Total 10–20,000 CD4^+^ cells were analyzed per group. Control peptide: KQFK peptide.

Our results demonstrate that the KRFK peptide can activate latent TGF-β and change DC phenotype and also act as a cross conjunctival epithelial barrier to reach underlying DCs. These both results make KRFK peptide a good candidate to modulate ocular surface DCs and therefore the systemic immune response induced by them. In TSP-1-deficient mice, antigen bearing DCs from conjunctiva that migrate to cervical lymph nodes were detectable within 3 h of antigen application [16]. When compared to WT mice, lymph nodes in TSP-1-deficient mice contain increased frequency of Th1 (CD4^+^ IFN-γ^+^), Th17 (CD4^+^ IL-17^+^), and reduced frequency of Tregs (CD4^+^ Foxp3^+^). To evaluate whether the topical application of KRFK peptide can alter such a systemic inflammatory immune response, TSP-1-deficient mice were treated with KRFK or inactive control KQFK prior to the onset of ocular surface inflammation (see Section 4.5 for details). At the end of the study period, cells from their cervical lymph nodes were collected. The frequency of inflammatory Th1, Th17, and regulatory Treg subsets were analyzed by flow cytometry. As shown in Figure 3, while a reduced frequency of Th1 and Th17 effectors was noted in KRFK-treated TSP-1-deficient mice as compared to the control group, the frequency of Tregs was increased. These results clearly support the ability of topically applied KRFK peptide to alter the systemic inflammatory immune response.

### 2.4. Topically Administered KRFK Peptide Prevents the Development of Chronic Ocular Inflammation Associated Signs in TSP-1-Deficient Mice

**Figure 4:**
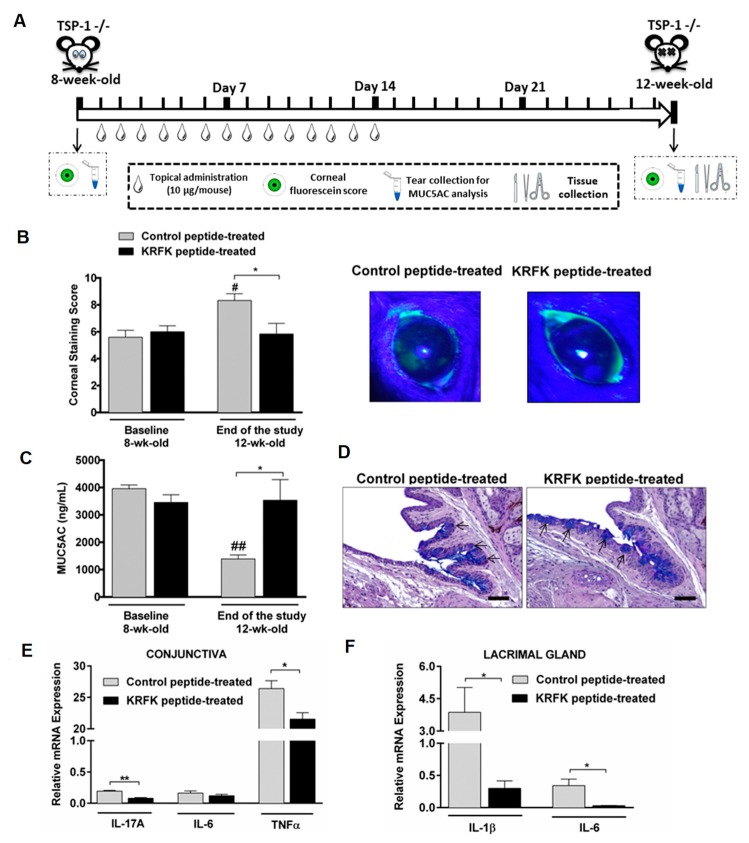
Topically administered KRFK peptide effectively prevents ocular surface inflammatory changes in TSP-1-deficient mice. (**A**) Schematic showing the experimental set up used for the in vivo treatment study. (**B**) Corneal staining scores at the baseline and at the end of the study (left) and representative images of corneal fluorescein (green) staining at the end of the study (right). (**C**) Tear MUC5AC levels, as determined by ELISA at the baseline and at the end of the study period. (**D**) Representative images of PAS/AB-stained conjunctival tissue sections at the end of the study showing filled goblet cells stained in blue (indicated by arrows). (**E**) Inflammatory cytokine mRNA expression in conjunctival and (**F**) lacrimal gland tissues as determined by real time RT-PCR at the end of the study period. Statistically significant differences between samples are indicated with asterisks and compared to the baseline are indicated with hashes (*, # *p* ≤ 0.05 and **, ## *p* ≤ 0.01). Scale bar: 50 µm.

TSP-1-deficient mice develop chronic ocular inflammation that is associated with Sjögren’s syndrome progressively as they age. Well-established signs of ocular surface inflammation are evident at twelve weeks of age. These include the disruption of corneal epithelial barrier integrity, reduced tear mucin levels, and the expression of inflammatory cytokines in cornea, conjunctiva, and lacrimal gland tissues [6,16,17,18,19]. When considering the ability of KRFK to alter the induction of the systemic immune response, here we evaluate its ability to prevent disease progression in TSP-1-deficient mice in a proof-of-concept study. For this, we initiated topical application of KRFK or the inactive control peptide KQFK in eight weeks old mice for a period of two weeks. We then compared signs of ocular surface inflammation two weeks later when mice reached twelve weeks of age, as depicted in the experimental design in Figure 4A.

Corneal barrier integrity was assessed by scoring corneal fluorescein staining before initiating the topical peptide application (baseline—eight weeks of age) and at the end of the study period (twelve weeks of age). As shown in Figure 4B, significantly increased corneal staining score is detectable in control peptide treated mice at twelve weeks of age as compared to baseline that is consistent with the progressive disease development. However, in the KRFK-treated group, such progression is prevented, as the corneal staining score is significantly reduced when compared to that detected in the control group.

Levels of soluble mucin (MUC5AC), derived from goblet cells, in tears correlate with the conjunctival inflammation in TSP-1-deficient mice [16]. Therefore, we evaluated tear MUC5AC levels in KRFK and control peptide-treated mice. Similar to corneal staining score, a progressive decline in tear MUC5AC level was detected at twelve weeks of age as compared to baseline in control peptide-treated mice. Such a decline was prevented in KRFK-treated mice and tear MUC5AC levels were significantly increased in these mice as compared to control peptide-treated mice at twelve weeks of age (Figure 4C). These results were further confirmed by comparing the number of PAS/AB stained filled goblet cells. As shown in Figure 4D, a higher number of filled goblet cells was detected in conjunctiva that were derived from KRFK-treated mice as compared to control peptide-treated mice.

The expression of inflammatory cytokines was assessed in the conjunctiva and lacrimal gland tissues at the end of the study. As shown in Figure 4E,F, significantly reduced expression of inflammatory cytokines was detected in tissues that were harvested from KRKF-treated mice as compared to the control group. Together, these results support the ability of KRFK to prevent disease progression in TSP-1 deficient mice.

### 2.5. Topically Administered KRFK Peptide Does not Induce Fibrotic Changes in the Ocular Surface of TSP-1-Deficient Mice

**Figure 5:**
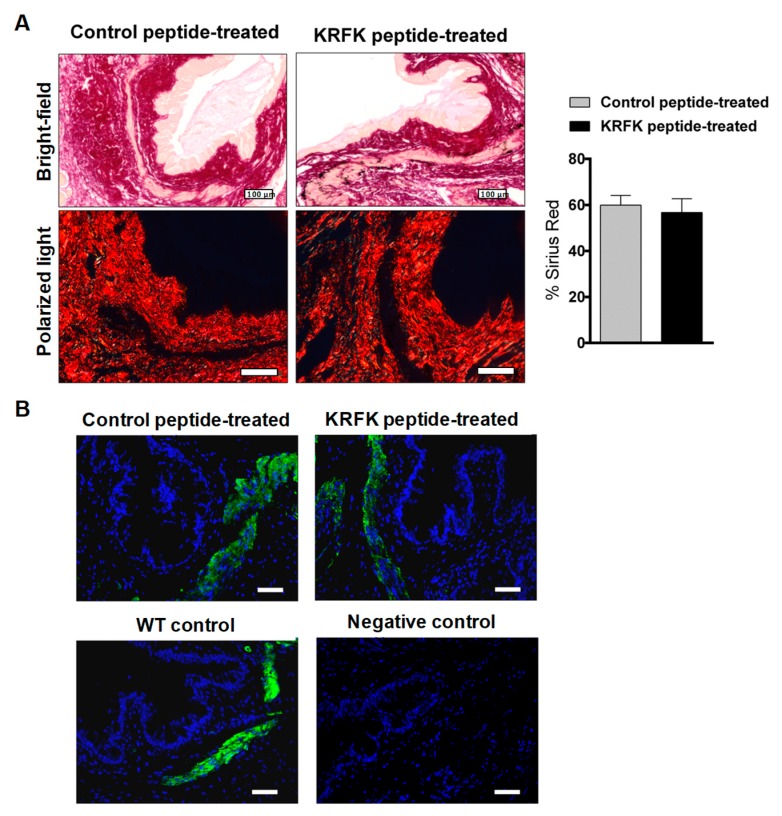
Topically administered KRFK peptide does not induce fibrotic changes in TSP-1-deficient conjunctiva. (**A**) Representative micrographs of Sirius red-stained ocular tissue sections obtained by bright-field and polarized light microscopy at the end of the study. Quantitative evaluation of Sirius red staining (*n* = 6 sections/group) is presented in the bar graph (right). (**B**) Representative micrographs of the immunolocalization of α-smooth muscle actin (α-SMA) (green) in TSP-1-deficient mice at the end of the study period and in age-matched WT control ocular tissue sections. Nuclei were stained with Hoechst dye (blue). Negative control includes omission of primary antibody. Scale bar: 50 µm, otherwise indicated.

As the RFK sequence activates latent TGF-β [15], and activated TGF-β mediates ocular fibrotic processes under a pathological environment [20,21], we next determine whether KRFK administration leads to fibrotic changes in the ocular surface tissue. Increased collagen deposition and an excess of myofibroblast cell type are common indicators of tissue fibrosis. To evaluate collagen content, Sirius red staining was performed on conjunctiva tissues that were harvested at the end of the study period, while the expression of α-SMA was assessed as a myofibroblast marker. There was no significant difference in collagen content (identified as the pink red staining in Sirius red stained tissue sections) of conjunctiva harvested from KRFK vs. control peptide treated groups (Figure 5A). Representative micrographs of α-SMA immunostaining of conjunctiva tissues show positive α-SMA, as identified by pale yellow staining in Sirius red-stained tissue sections. Such localization in the smooth muscle areas is seen in normal tissues and during the repair process that involves fibroblast to myofibroblast differentiation. In the conjunctival stromal area, similar α-SMA staining was detected in tissue derived from both groups of TSP-1-deficient mice. Similar staining was also detected in the conjunctiva of age-matched WT mice (Figure 5B), thus confirming the absence of excessive myofibroblasts. Overall, these observations rule out the development of fibrosis in the conjunctiva as a result of KRFK administration.

## 3. Discussion

Thrombospondin-1 is a critical immune modulator at the ocular mucosal surface [5,16]. This role is consistent with the predominance of TGF-β2 expression by ocular surface epithelial cells, both in mice and humans [5,22,23], and the ability of TSP-1 to efficiently activate this isoform of TGF-β [15]. Moreover, TSP-1 deficiency in mice results in a development of chronic ocular surface inflammation [6]. Similarly, humans with a TSP-1 variant corresponding with the reduced TSP-1 expression in the ocular surface, epithelial cells are susceptible to developing chronic ocular surface inflammation [7]. Previously, in different mouse models with ocular surface inflammation we detected increased staining for TGF-β, presumably latent and biologically inactive form [8]. In this study, we used a well-characterized TGF-β-activating peptide that was derived from TSP-1 (KRFK) to determine its ability to restore ocular surface immune modulation and prevent the development of chronic ocular surface inflammation in TSP-1-deficient mice.

Spontaneously developed chronic ocular surface inflammation in TSP-1-deficient mice progresses with age [6,16,18]. While normal at birth, these mice gradually develop chronic ocular surface inflammation, which is fully established by twelve weeks of age with quantifiable clinical signs, like the disrupted corneal epithelial barrier integrity and the secretory dysfunction of mucin secreting conjunctival goblet cells. These changes are accompanied by histologically detectable loss of goblet cells and inflammatory infiltration of the conjunctiva and the lacrimal gland. Systemically, a peripheral imbalance in Treg and pathogenic Th17 effectors is noted in lymphoid tissues [6,16,17]. The homing of inflammatory lymphocytes to ocular surface plays a central role in the effector phase of the chronic condition [24,25]. In our experiments, systemic changes that were noted after KRFK treatment correlate with subsequently reduced clinical symptoms of ocular surface inflammation at twelve weeks of age. Overall, our results demonstrate KRFK as a potential therapeutic option to prevent the development of chronic inflammatory manifestations of the ocular surface.

We have previously reported that APCs exposed to TGF-β counter inflammation by inducing T regulatory effectors [4]. Such tolerogenic ability is partially facilitated by their expression of TSP-1. These observations support a loss of tolerance induction by TSP-1-deficient APCs and consequential detection of inflammatory effectors in the lymphoid tissues of TSP-1-deficient mice [4,6]. Now, our in vitro results demonstrate that although TSP-1-deficient BMDCs express endogenous latent TGF-β they are unable to activate it, like WT BMDCs. In our experiments, the TGF-β-activating sequence in KRFK effectively activated latent TGF-β produced by TSP-1-deficient BMDCs. As a result, a reduced expression of the costimulatory molecule, CD80, was detected in KRFK-treated BMDCs. However, a similar effect was not observed on MHC class II expression, which is possibly due to a need for the longer exposure time and/or higher concentration of the peptide. Overall, our results indicate that TSP-1 derived peptide KRFK can help to promote a tolerogenic phenotype of TSP-1- deficient DCs by facilitating the activation of their latent TGF-β.

These in vitro findings suggest that the TSP-1-derived KRFK peptide could contribute to the development of regulatory immunity via modulating local ocular APCs if applied topically to the eye. However, typically, access to such APCs is limited by ocular surface barriers and the physicochemical properties of any drug to be used. Generally, factors that contribute to poor drug absorption include its high molecular weight (>500 g/mol), more than five hydrogen bonds and more than 10 hydrogen bond acceptors in the drug structure in addition to a high lipophilicity. The KRFK peptide has none of these properties, although due to its small length (4 aa), a low stability and short half-life could be expected [26]. Nonetheless, some studies have demonstrated its functionality without structural modification [14,27,28]. Moreover, we have reported KRFK permeability in vitro across conjunctival epithelial culture [29]. In these in vitro experiments, we were able to successfully detect a steady passage of KRFK peptide across conjunctival epithelium during the evaluated intervals of time (15 min to 3 h) [29]. However, in vivo, a lower bioavailability of the peptides may result from additional biopharmaceutical barriers, such as tear drainage and proteolytic activity of the tear film [30]. In this study, our results indicate that, after topical application, the KRFK peptide is retained in the ocular surface up to 1 h. We have previously reported that, after topical application of an antigen in TSP-1-deficient mice, DCs carrying this antigen are detected in the draining cervical lymph nodes within 3 h of antigen application and that this migration is inhibited by administration of TSP-1 [16]. Given such kinetics of DC migration in TSP-1-deficient mice, we believe that KRFK retention in ocular surface tissue is of sufficient duration to influence the phenotype of local APCs in vivo. Together, our results supported a strong potential of KRFK peptide to influence systemic immune response against ocular surface derived antigens that are involved in the development of chronic ocular surface inflammation.

Based on our in vitro findings and the ocular surface retention of KRFK peptide, we opted to assess its effect on the systemic immune response in TSP-1-deficient mice. The effect of KRFK peptide on TSP-1-deficient BMDCs in vitro is consistent with the observed decrease in effector Th1 and Th17 populations, together with an increment in Treg population in cervical lymph nodes of KRFK-treated TSP-1-deficient mice. By activating latent TGF-β available in the ocular surface tissue, it is likely that KRFK helps to maintain tolerogenic phenotype of local DCs, thereby preventing the induction of inflammatory effectors.

Together with changes observed in T cell populations after KRFK treatment, our results demonstrate that KRFK peptide also prevents the development of clinical signs, like the disrupted corneal barrier and the reduced tear MUC5AC levels in TSP-1 deficient mice. Changes in MUC5AC levels in our experiments are consistent with observations reported in other mucosal surfaces demonstrating TGF-β-induced MUC5AC expression [23,31]. Furthermore, Th1 cytokines, IFN-γ and TNF-α, are known to inhibit mucin secretion by goblet cells and induce their apoptosis, contributing to their loss during ocular surface inflammation and their neutralization restores goblet cell numbers in the conjunctiva [32,33]. These findings are consistent with the downregulated expression of TNF-α mRNA noted in our experiments in conjunctival tissues after KRFK treatment. Additionally, prevention of the disrupted corneal barrier in KRFK-treated mice also correlates with the observed reduced expression of IL-1β and IL-6, cytokines that are frequently up-regulated in the tears of patients with ocular surface inflammatory diseases [7,34,35,36,37,38]. Specifically, IL-1β has been highlighted as a key player in different corneal epithelium-related pathologies [39,40,41]. Collectively, our data from ocular surface tissues indicate a clear efficacy of KRFK, a TSP-1-derived peptide, in preventing the development of chronic ocular surface inflammation. This effect is achieved in the absence of any fibrotic changes resulting from TGF-β activation, as ruled out by examined markers of tissue fibrosis.

## 4. Materials and Methods

### 4.1. Mice

The use of animals in this study was in accordance with the recommendations of ARVO Statement for the Use of Animals in Ophthalmic and Vision Research. The Institutional Animal Care and Use Committee at Boston University School of Medicine (Boston, MA, USA) approved animal studies described in this manuscript (Protocol AN-15400, 30 September 2016).

TSP-1-deficient mice, on C57BL/6 background that was originally purchased from The Jackson Laboratory, Bar Harbor, ME, were bred in a pathogen-free facility. Six to twelve-weeks-old C57BL/6 (H-2b) mice (Charles River Laboratories, Wilmington, MA, USA) were used as wild type (WT) controls in this study.

### 4.2. Antibodies and Reagents

All of the materials used were purchased from Sigma-Aldrich (Saint Louis, MO, USA), unless otherwise indicated. Recombinant GM-CSF was purchased from BioLegend (San Diego, CA, USA) and Ionomycin from EMD Millipore (Burlington, MA, USA). TSP-1 derived peptide KRFK and inactive control peptide Lys-Gln-Phe-Lys (KQFK) were synthesized by Bionova (Madrid, Spain). Complete RPMI-1640 was prepared by adding 10 mM HEPES, 0.1 mM NEAA, 1 mM sodium pyruvate, 100 U/mL Penicillin, 100 mg/mL streptomycin, 200 mM l-glutamine (all from Lonza, Basel, Switzerland), and 10% fetal bovine serum (Atlanta Biologicals, GA, USA). Anti-CD4-PE-Cy-5 (clone RM4-5) was purchased from BioLegend (San Diego, CA, USA), anti-IFN-γ-FITC (clone XMG1.2), anti-IL-17-PE (clone eBio17B7), and anti-Foxp3-PE-Cy5 (clone FJK-16s) were purchased from eBioscience/Thermofisher scientific (San Diego, CA, USA). Anti-SMA (clone 1A4) was purchased from Abcam (Cambridge, MA, USA). Alexa Fluor 488-conjugated secondary antibodies were purchased from Thermofisher scientific (Waltham, MA, USA).

### 4.3. Bone Marrow-Derived Dendritic Cell Cultures

Bone marrow cells were harvested from WT or TSP-1-deficient mice and cultured in complete RPMI-1640 medium with 20 ng/mL of murine recombinant granulocyte-macrophage colony stimulating factor (GM-CSF) for 6 d, as described previously [16,42]. Dendritic cells from these cultures were treated with 50 μM of TSP-1-derived (KRFK) or control (KQFK) peptides that are based on a previous study [14], in serum-free RPMI-1640 medium for a period of 24 h. At the end of the culture period, supernatants and cell lysates were collected for further analysis.

### 4.4. TGF-β Bioassay

Total and active amounts of TGF-β were measured using MFB-F11 cells, a fibroblasts cell line derived from TGF-β-knockout mice that is stably transfected with a secreted alkaline phosphatase reporter gene [43]. After initial 24 h culture in serum-free DMEM, cells were cultured with BMDC supernatants for 24 h. Culture supernatants were acid-treated (1N HCl) to activate latent TGF-β. Active TGF-β levels were determined in both untreated and acid-treated supernatants by detecting SEAP activity using the Great EscAPe SEAP Reporter system 3 (Clontech, Mountain View, CA, USA), as per the manufacturer’s instructions. A recombinant TGF-β2 was used to generate the standard curve. Active TGF-β content was determined in samples collected from 3 independent experiments.

### 4.5. Topical FITC-Conjugated TSP-1-Derived Peptide KRFK Administration and In Vivo Localization

To determine an approximate retention time of the KRFK peptide in ocular surface tissues after its topical application, a FITC-conjugated KRFK peptide was used. This labeled-peptide (5 µg) was administered topically in a total 5 μL volume per eye to WT mice. Eyeballs were harvested at 2 different intervals (1 and 3 h after administration) and were embedded in optimal cutting temperature (OCT) compound and then flash-frozen in liquid nitrogen. Frozen sections of 5 µm thickness were cut and stained with Hoechst 33342 dye to visualize cell nuclei. Sections were examined with a ×20 objective of a FSX100 Olympus fluorescence microscope (Center Valley, PA, USA).

### 4.6. Treatment Set up and Disease Monitoring—Proof of Concept Study

TSP-1-deficient mice were used as a mouse model of chronic ocular surface inflammation [6]. Female mice at eight weeks of age (prior to the onset of ocular surface inflammation) were treated topically with 5 μL of KRFK or control (KQFK) peptides at 1 μg/μL, once a day during two weeks.

Disease progression was monitored by assessing corneal fluorescein staining and tear MUC5AC content (*n* = 6 eyes/group) before initiating and after completing the treatment. Corneal fluorescein staining and pilocarpine-induced tear collection were performed, as previously described [6]. Briefly, to detect corneal fluorescein staining application of sodium fluorescein (1%) to the mouse eye was followed by PBS washes and the evaluation of corneal staining using a slit lamp microscope and a cobalt blue light. Punctate staining was graded based on the standardized National Eye Institute grading system [44]. Pilocarpine-induced tears were collected and MUC5AC content determined using ELISA kit (TSZ ELISA, Waltham, MA, USA). Results are reported as MUC5AC in ng/mL of tear volume. Ocular tissues and cervical lymph nodes were collected at the study endpoint for cellular and molecular analyses.

### 4.7. Intracellular Cytokine Staining and Flow Cytometry

Cervical lymph nodes from peptide-treated TSP-1-deficient mice were harvested and single cell suspensions were prepared. Cells were then stimulated with phorbol 12-myristate 13-acetate (PMA, 50 ng/mL) and Ionomycin (2 μg/mL) for 4 h with Brefeldin A (1×) added for the final 3h. Cells were stained with fixable viability dye eFluor™ 780, fixed and permeabilized using intracellular staining kit (eBioscience, San Diego, CA), followed by staining for cell surface CD4 and intracellular IFN-γ, IL-17, and Foxp3 using fluorochrome conjugated antibodies. Stained cells were analyzed using BD LSRII Flow Cytometer (BD Bioscience, San Jose, CA, USA) at Boston University Core Services. Data was analyzed further using FlowJo v9.4.10 software (Tree Star, Inc. Ashland, OR, USA).

### 4.8. Tissue Processing and Histopathological Analysis

Ocular tissues that were harvested from WT and peptide-treated-TSP-1-deficient mice were fixed in 4% paraformaldehyde solution, embedded in paraffin, and sliced in sagittal sections of 5 µm thickness.

Hematoxylin and eosin (H&E) stained sections were used to evaluate tissue morphology. Conjunctival and corneal epithelial thicknesses were measured to consider the possible differences among experimental groups. Representative micrographs were taken using ×10 objective.

Tissue sections stained with Periodic acid–Schiff and alcian blue (PAS/AB) were used to count filled-goblet cells in the superior and inferior bulbar and tarsal conjunctivas, in a masked fashion by two independent trained observers. Sagittal sections from the center of the eye were used as representative of the whole conjunctiva to control for the variations in goblet cell density over the ocular surface. Representative micrographs were taken using ×10 objective.

To evaluate collagen deposition and fibrosis in the ocular tissues, sections were stained with the Sirius red dye (aqueous solution of picric acid with 0.1% of Sirius red for 1 h). Stained tissue sections were evaluated under a light microscope with ×10 or ×40 objective with polarized light. Micrographs that were taken using ×10 objective were analyzed using ImageJ software v. 1.49 (National Institutes of Health, Bethesda, MD, USA). Percentage of Sirius red staining was calculated.

### 4.9. Immunofluorescence Staining

α-smooth muscle actin (α-SMA) was immunodetected in paraffin-embedded ocular tissue sections of peptide treated-TSP-1 deficient mice. For α-SMA detection, the tissue sections were incubated with 0.01% trypsin for antigen retrieval, followed by the blocking buffer composed of 5% goat serum and 0.3% Triton X-100 at room temperature (RT) for 1 h. Sections were then incubated with primary antibody (α-SMA) at 2.5 µg/mL in blocking buffer at 4 °C overnight. After rinsing in PBS, sections were incubated with Alexa Fluor-conjugated secondary antibody at 10 µg/mL in PBS at RT for 1 h. Primary antibody was omitted in Negative controls and Hoechst 33342 dye was used to counterstain nuclei in blue. Stained sections were examined using the fluorescence microscope Leica DMI 6000B and the LAS AF Lite software from Leica Microsystems (Wetzlar, Germany). An exposure time, gain, and intensity of the camera were stablished and representative micrographs were taken. The specificity of the primary antibody was previously confirmed using murine ocular tissues.

### 4.10. Real Time RT-PCR Analysis

Total RNA was isolated from BMDCs and from conjunctival and lacrimal gland tissues that were harvested from TSP-1-deficient mice using TRIzol Reagent (Life Technologies, Carlsbad, CA, USA) and cDNA was synthesized using the SuperScript^®^ VILO™ cDNA Kit (Life Technologies).

Real time PCR was performed using cDNA, primers and SYBR Green PCR Master Mix (Life Technologies) with a thermal profile: 95 °C (180 s) and 40 cycles of 95 °C (15 s), 55 °C (30 s), and 72 °C (40 s). The glyceraldehyde-3-phosphate dehydrogenase (GAPDH) signal was used to normalize values. All reactions were performed in triplicate and included a non-template control. The sequences of primer pairs used are provided in Table 1. To verify the specificity of the amplification reaction, a melting curve analysis was performed. Fluorescence signal that was generated at each cycle was analyzed using system software. The threshold cycle values were used to determine the relative quantification of gene expression with glyceraldehyde-3-phosphate dehydrogenase as a reference gene.

### 4.11. Statistical Analysis

Results are presented as mean ± standard error of the mean (SEM). Statistical significance between different conditions was assessed using the Student´s t-test with a Welch´s correction applied in case of significantly different variances (F test). To compare more than two conditions, a one-way ANOVA followed by Tukey test was performed, unless there was significant heterogeneity of variance (Levene´s test), in which case a robust test (Brown-Forsythe test) was performed, followed by the Games–Howell test. A value of *p* ≤ 0.05 was considered to be significant.

## 5. Conclusions

To conclude, our study demonstrates that the topical administration of TGFβ-activating KRFK peptide offers an advantage in the efficacy without undesirable profibrotic effects. This study underlines the effect that TSP-1 exerts in orchestrating the regulation of inflammation via TGF-β activation and demonstrates that KRFK peptide, as derived from TSP-1, can successfully prevent the development of chronic ocular inflammation.

## Figures and Tables

**Table 1:** Sequences of the primer pairs used.

Gen	Sequence for forward Primer	Sequence for Reverse Primer
MHC II ^1^	5’-AGG GCA TTT CGT GTA CCA GTT-3’	5’-GTA CTC CTC CCG GTT GTA GAT-3’
CD80	5’-GAA TTA CCT GGC ATC AAT ACG-3’	5’-CTT AAT GGT GTG GTT GCG AGT C-3’
IL-6 ^2^	5’-AGT CAA TTC CAG AAA CCG CTA TGA-3’	5’-TAG GGA AGG CCG TGG TTG T-3’
IL-1β ^2^	5’-TCT GAA GCA GCT ATG GCA ACT GTT-3’	5’-CAT CTT TTG GGG TCC GTC AAC T-3’
TNFα ^3^	5’-GGC CTC CCT CTC ATC AGT TCT ATG-3’	5’-GTT TGC TAC GAC GTG GGC TAC A-3’
IL-17A ^2^	5’-AGT GAA GGC AGC AGC GAT CAT-3’	5’-CGC CAA GGG AGT TAA AG-3’
GAPDH ^4^	5’-GAACGTGAAGGTCGGAGTCAAC-3’	5’-CGTGAAGATGGTGATGGGATTTC-3’

^1^ MHC II: major histocompatibility complex class II; ^2^ IL: interleukin; ^3^ TNFα: tumor necrosis factor-α; ^4^ GAPDH: glyceraldehyde-3-phosphate dehydrogenase.

## Abbreviations

AB	alcian blue
SBE-SEAP	Smad-binding elements coupled to a secreted alkaline phosphatase reporter gene
SMA	smooth muscle actin
